# Nonalcoholic fatty liver disease is associated with coronary artery calcium score in diabetes patients with higher HbA1c

**DOI:** 10.1186/s13098-015-0025-4

**Published:** 2015-03-28

**Authors:** Min-Sun Kwak, Jeong Yoon Yim, Donghee Kim, Min Jung Park, Seon Hee Lim, Jong In Yang, Goh Eun Chung, Young Sun Kim, Sun Young Yang, Mi Na Kim, Chang-Hoon Lee, Jung-Hwan Yoon, Hyo-Suk Lee

**Affiliations:** Department of Internal Medicine, Healthcare Research Institute, Healthcare System Gangnam Center, Seoul National University Hospital, 39FL., Gangnam Finance Center 737, Yeoksam-Dong, Seoul, 135-984 Gangnam-Gu Korea; Division of Pulmonary and Critical Care Medicine, Department of Internal Medicine, Seoul National University College of Medicine, Seoul, Korea; Department of Internal Medicine and Liver Research Institute, Seoul National University College of Medicine, Seoul, Korea

**Keywords:** Nonalcoholic fatty liver disease, Coronary artery calcium score, Diabetes, HbA1c

## Abstract

**Background:**

In patients with diabetes, studies investigating the association between nonalcoholic fatty liver disease (NAFLD) and coronary artery calcium score (CACS) have shown conflicting results. The aim of this study was to evaluate the association between NAFLD and CACS in diabetic patients.

**Methods:**

This is the cohort study performed in Seoul National University Hospital Gangnam Healthcare Center. NAFLD was defined as cases with the typical ultrasonographic findings without excessive alcohol consumption, medications causing hepatic steatosis or other chronic liver diseases. CACS was evaluated using the Agatston method. Diabetes was defined as cases with fasting serum glucose ≥ 126 mg/dl, glycated hemoglobin (HbA1c) ≥ 6.5%, or those taking anti-diabetic medications. Multivariate linear regression analyses were performed with use of the interaction term of NAFLD × glycemic level and other confounders of CACS such as age, sex, hypertension, body mass index, waist circumference, HDL cholesterol and triglyceride.

**Results:**

A total of 213 participants with diabetes were included in the study. As 77 subjects (36.2%) had CACS 0, causing left sided skewness, CACS was analyzed after log transformation to Ln (CACS + 1). A statistically significant interaction was observed between NAFLD and HbA1c ≥ 7% (*P* for interaction = 0.014). While NAFLD was not associated with CACS in the group with HbA1c < 7% (*P* = 0.229), it was significantly associated in the group with HbA1c ≥ 7% (*P* = 0.010) after adjusting for covariates in multivariate analyses.

**Conclusions:**

This study demonstrated an effect modification of glycemic level on the association between NAFLD and CACS. NAFLD was independently associated with CACS only in diabetes patients with higher HbA1c, after adjustment for confounders.

## Background

Nonalcoholic fatty liver disease (NAFLD) is the most common cause of chronic liver disease, with an estimated prevalence of 20-30% in the general population. In people with obesity or type 2 diabetes, the prevalence of NAFLD further increases to 70-90% [1,2]. NAFLD is closely associated with metabolic syndrome, abdominal obesity, and insulin resistance [3]. And NAFLD is also associated with an increased risk of cardiovascular disease, independent of the other metabolic syndrome risk factors [4,5]. Coronary artery calcification is a well-established surrogate marker of subclinical coronary artery disease, and is known to reflect the atherosclerotic burden and risk of cardiovascular disease outcomes [2,6]. Several previous studies demonstrated the association of coronary artery calcium score (CACS) with NAFLD, after adjustment for the other related factors [2,7-11].

Meanwhile, NAFLD is closely associated with diabetes [3]. There have been conflicting results about the association between NAFLD and cardiovascular disease in patients with diabetes. Some studies indicated that NAFLD is associated with a higher prevalence of coronary artery disease in type 2 diabetic patients [12,13]. In contrast, another study showed no significant association between NAFLD and CACS in patients with diabetes [14]. In addition, studies evaluating the association of NAFLD and carotid intima media thickness, another surrogate marker for cardiovascular disease, have also reported conflicting results about the association between carotid intima media thickness and NAFLD in diabetic patients [14,15].

Therefore, the aim of this study was to evaluate the association between NAFLD and CACS, specifically in diabetic patients, after adjustment for other known risk factors of cardiovascular disease.

## Methods

### Study population

**Figure 1:**
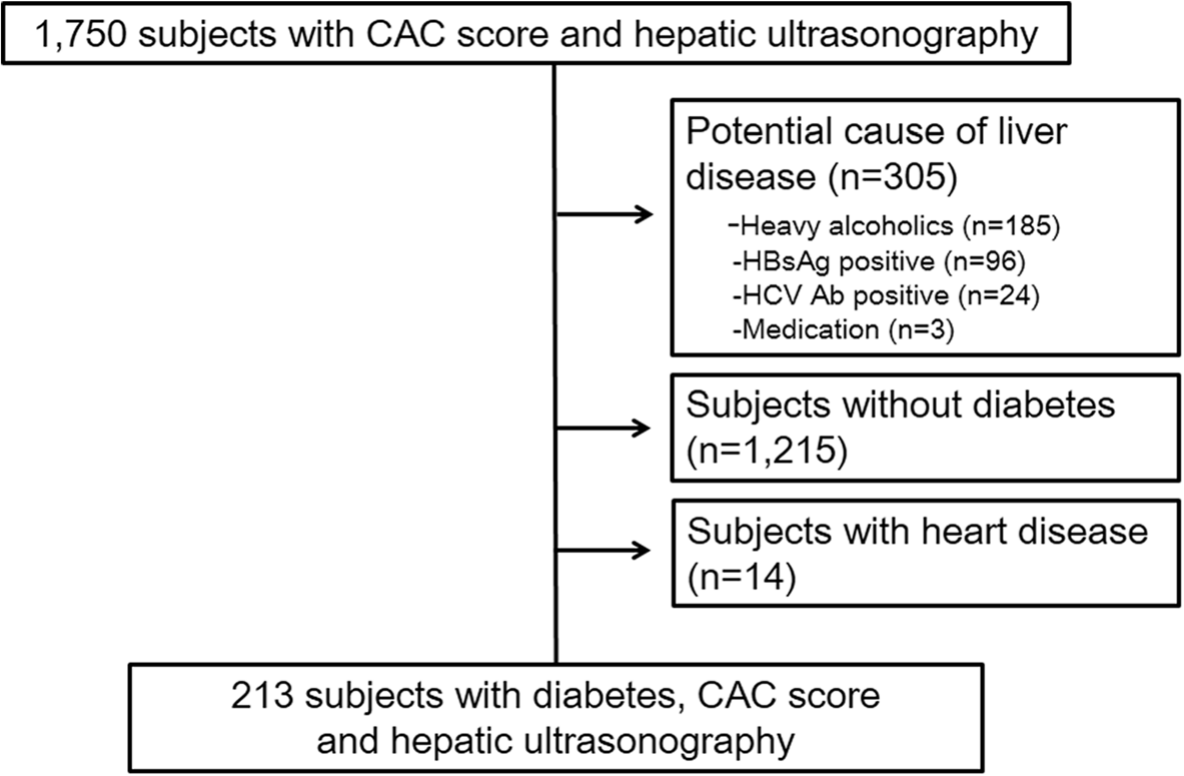
**Flowchart showing details of inclusion and exclusion of the subjects.**

Patients who visited the Seoul National University Hospital Gangnam Healthcare Center for health screening between July 2011 to July 2012 and performed coronary calcium-scoring computed tomography, hepatic ultrasonography, and baseline laboratory exams were initially included in the study. Subjects with other potential causes of chronic liver disease were excluded as follows: subjects with excessive alcohol consumption (>30 g/day for men and > 20 g/day for women), with hepatitis B virus (determined by the presence of the hepatitis B surface antigen), with the hepatitis C virus (determined by the presence of the hepatitis C antibody), or with some other history of hepatitis, as identified using a detailed medical history and a questionnaire (primary biliary cirrhosis, Wilson’s disease, hemochromatosis, and autoimmune hepatitis). Subjects taking medications that can account for steatosis (e.g. tamoxifen, amiodarone, methotrexate) during the previous year were also excluded. Among them, we selected the patients who were diagnosed with diabetes. The presence of diabetes was defined as having either a fasting serum glucose level equal to or greater than 126 mg/dL, glycated hemoglobin (HbA1c) ≥ 6.5% or consuming medication for diabetes. Participants who had a history of heart attack, coronary artery disease including acute myocardial infarction, angina, or congestive heart failure were also excluded. Figure 1 shows flowchart of the inclusion and exclusion of the patients.

The study protocol conformed to the ethical guidelines of the 1975 Declaration of Helsinki and was approved by the Institutional Review Board of Seoul National University Hospital (H-1406-040-585).

### Clinical, laboratory and radiological assessments

Each participant completed a questionnaire about past medical history, and performed an anthropometric assessment, laboratory tests and radiologic tests on the same day. Height and body weight were measured using a digital scale while wearing a light gown. The body mass index (BMI) was calculated as follows: BMI = weight (kg)/height squared (m^2^). Waist circumference (WC) was measured with a tape measure to the nearest millimeter at the midpoint between the lower costal margin and the iliac crest by a well-trained examiner. Systolic and diastolic blood pressures were measured twice on the same day in the seated position, and the mean values were used for analysis. The presence of hypertension was defined as having systolic blood pressure over 140 mmHg or diastolic blood pressure over 90 mmHg more than twice or as taking anti-hypertensive medication. Current smokers were defined as those who had smoked at least one cigarette per day during the previous year. Ex-smokers were defined as thosewho used to smoke cigarettes regularly.

Laboratory examinations included serum aspartate aminotransferase (AST), alanine aminotransferase (ALT), gamma-glutamyl transpeptidase (GGT), triglyceride, high-density lipoprotein cholesterol, low-density lipoprotein cholesterol, uric acid, fasting glucose, HbA1c, hepatitis B surface antigen, and antibody to hepatitis C virus. Blood samples were collected from all participants before 10 AM after a 12-h overnight fast. All of the biochemical determinations were carried out in the same laboratory using standard laboratory methods.

### Diagnosis of fatty liver by ultrasonography

Hepatic ultrasonographic examinations were performed by experienced radiologists who were blinded to the clinical and laboratory details of the participants at the time of the procedure. The diagnosis of fatty liver was performed by ultrasonography (Acuson, Sequoia 512, Siemens, Mountain View, CA) using hepatorenal echo contrast, liver brightness, deep attenuation, and vascular blurring [16].

### Measurement of CACS by multidetector CT

A Scanning of the coronary artery was performed using a 16-row multi-slice CT scanner (Sensation 16; Siemens Medical Systems, Erlangen, Germany). CAC scans were acquired using the standard procedure of the prospective echocardiography-triggered scan acquisition with a tube voltage of 120 kV, and 110 effective mAs with a 200 mm field of view [17]. Data were reconstructed to a 3-mm slice thickness at a −400 ms acquisition window. The CACS was then calculated using a CT software program (Rapidia 2.8; INFINITT, Seoul, Korea) according to the method described by Agatston et al. [18].

### Statistical analysis

The chi-square test was used for categorical variables, while Student’s t-test and the Mann–Whitney U test were used for continuous variables to identify the differences between the subjects with and without NAFLD. The distribution of CACS was evaluated, and log transformation was considered in the case of left skewness. We attempted to evaluate if there were any effect modification by glycemic level in the relationship of NAFLD and CACS using the interaction term of HbA1c ≥ 7% × NAFLD. The HbA1c was divided at the level of 7%, as current guidelines specify HbA1c targets approximating or less than 7%, and the mean value of HbA1c level in this study is nearly 7% [19,20]. Considering collinearity, several covariates were selected and univariate linear regression analyses were performed to evaluate the association between Ln (CACS + 1) and the covariates. Age, sex, NAFLD, an interaction term of HbA1c ≥ 7% × NAFLD and other covariates obtaining a *P* value < 0.2 in the univariate analyses were included in the multivariate linear regression analysis. All statistical analyses were conducted using SPSS 19 (SPSS Inc., Chicago, IL, USA) and Stata 13.1 (StataCorp, College Station, Texas, USA). A two-tailed *P* value < 0.05 was considered statistically significant.

## Results

A total of 213 participants (184 males and 29 females, mean age 59 years) with diabetes were included in this study. The mean HbA1c level in the study population was 6.93% ± 0.89 with a fasting glucose level of 138.5 mg/dL ± 31.6. Among the diabetic patients, NAFLD was present in 86 subjects (40.4%). The baseline characteristics of the subjects with and without NAFLD are shown in Table 1. Seventy-seven subjects (36.2 %) had CACS 0. The median value of CACS was 11.3 (range, 0–2443.7) in the control group, and 44.2 (range, 0–1891.7) in the NAFLD group, showing left sided skewness. Therefore, we used a log-transformed new dependent variable: Ln (CACS + 1). We performed univariate linear regression to elucidate the association of Ln (CACS + 1) for covariates. Considering collinearity, we selected the variable of HbA1c ≥ 7% among HbA1c, HbA1c ≥ 7% and fasting glucose level. AST, ALT and GGT were also not evaluated in the regression analyses because of the collinearity to the variable NAFLD. Covariates included age, sex, hypertension, body mass index, waist circumference, high-density lipoprotein cholesterol and triglyceride. All these covariates could be considered as confounders in the association between NAFLD and CACS.

**Table 1:** **Baseline characteristics according to groups**

	**Control (n = 127)**	**NAFLD (n = 86)**	***P*** **-value**
Age, years old, median (range)	58 (36–80)	58 (38–75)	0.197
Male sex, n (%)	110 (86.6%)	74 (86.0%)	0.906
Hypertension, n (%)	61 (48.0%)	57 (66.3%)	0.009
Smoking, n (%)			0.298
Current or ex-smoker	82 (64.6%)	61 (70.9%)	
Unknown	11 (8.7%)	3 (3.5%)	
Exercise, n (%)			0.402
Regular exercise	21 (16.5%)	20 (23.3%)	
Unknown	35 (27.6%)	19 (22.1%)	
Body mass index, kg/m^2^, median (range)	24.1 (18.5-29.6)	26.2 (19.2-36.5)	<0.001
Waist circumference, cm, median (range)	87.5 (69.5-107)	92 (72.5-115)	<0.001
Metabolic syndrome, n (%)	45 (36.6%)	59 (68.6%)	<0.001
HbA1c, %, median (range)	6.60 (5.8-10.2)	6.8 (5.7-10.6)	0.166
HbA1c ≥ 7%, n (%)	45 (35.4%)	37 (43.0%)	0.264
Fasting glucose, mg/dL, median (range)	130 (84–252)	138 (84–307)	0.013
LDL cholesterol, mg/dL, median (range)	117.5 (58–268)	125.5 (51–220)	0.453
HDL cholesterol, mg/dL, median (range)	50 (29–89)	48 (32–67)	<0.001
Triglyceride, mg/dL, median (range)	92 (30–529)	135.5 (41–450)	<0.001
ALT, IU/L, median (range)	22 (8–64)	33 (11–161)	<0.001
AST, IU/L, median (range)	24 (11–112)	26.5 (13–202)	0.062
GGT, IU/L, median (range)	28.0 (8–266)	38.0 (12–243)	0.016
Uric acid, median (range)	5.7 (3–9.6)	5.8 (3.1-8.7)	0.253
CACS, median (range)	11.3 (0–2443.7)	44.2 (0–1891.7)	0.577

*Abbreviations*: *NAFLD* Nonalcoholic fatty liver disease, *AST* aspartate aminotransferase, *ALT* alanine aminotransferase, *GGT* gamma-glutamyl transpeptidase.

Ln(CACS + 1) was statistically significantly associated with increased age (*P* < 0.001), male sex (*P* = 0.027), hypertension (*P* = 0.035), and waist circumference (*P* = 0.020) in the univariate linear regression analyses (Table 2). HbA1c ≥ 7% (*P* = 0.051) showed marginal significance in the association with Ln(CACS + 1). In the multivariate linear regression models with adjustment of covariates with *P* < 0.2 from the univariate analyses, the interaction term HbA1c ≥ 7% × NAFLD was statistically significant. (*P* for interaction = 0.014). In addition, there was dose–response interaction between quartiles of HbA1c level and NAFLD in the association with CACS (*P* for interaction = 0.002).

**Table 2:** **Univariate linear regression analyses for the relationship with ln (CACS + 1)**

	**β-coefficient**	**95% CI**	***P*** **-value**
NAFLD	0.628	−0.091-1.347	0.087
Age	0.087	0.046-0.128	<0.001
Male sex	1.158	0.134-2.181	0.027
Hypertension	0.761	0.054-1.468	0.035
Body mass index	0.102	−0.022-0.227	0.107
Waist circumference	0.054	0.009-0.100	0.020
HDL cholesterol	−0.027	−0.062-0.009	0.139
Triglyceride	0.005	0.000-0.010	0.075
HbA1c ≥ 7%	0.720	−0.004-1.443	0.051

*Abbreviations*: *NAFLD* Nonalcoholic fatty liver disease, *CI* confidence interval.

**Figure 2:**
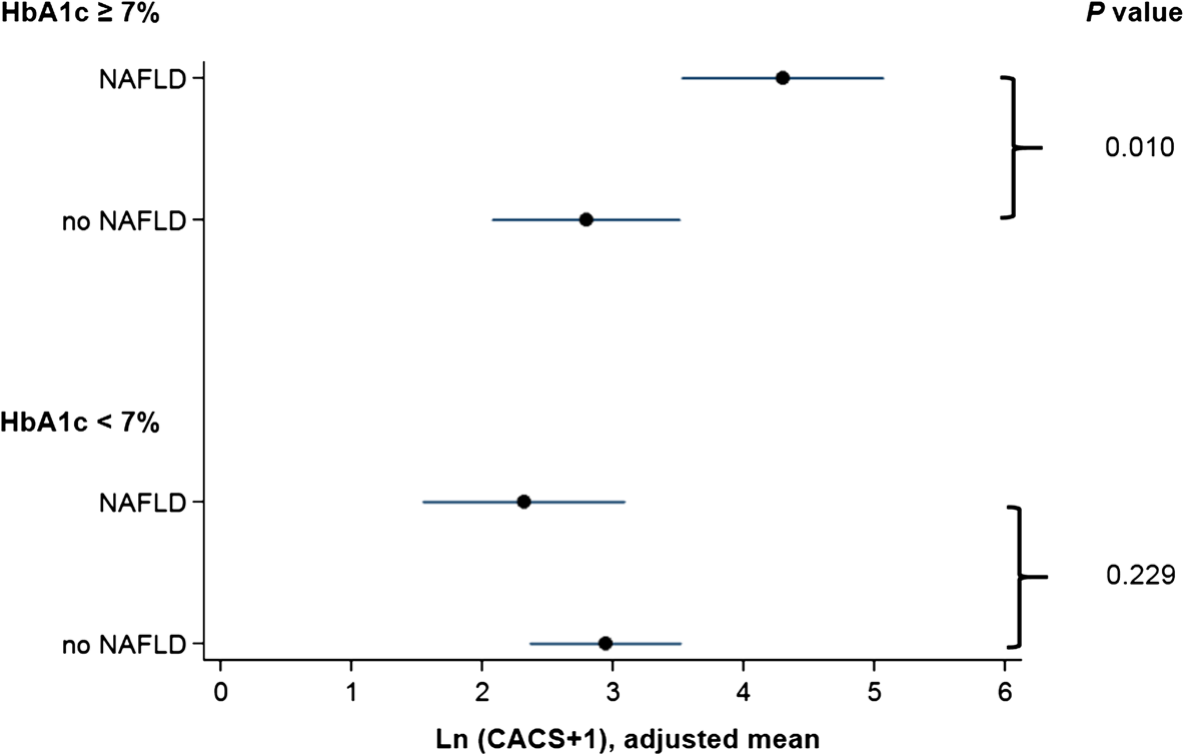
**Association of nonalcoholic fatty liver disease and coronary artery calcium score in diabetes patients with higher HbA1c.** In the group with HbA1c < 7%, NAFLD was not significantly associated with Ln (CACS + 1) (*P* = 0.229). However, NAFLD was independently associated with higher Ln (CACS + 1) in the group with HbA1c ≥ 7%, after adjusting for other covariates (*P* = 0.010).

Therefore, the multivariate linear regression analysis was analyzed separately according to the HbA1c level. In the group with lower HbA1c (HbA1c < 7%), NAFLD was not significantly associated with Ln (CACS + 1) (*P* = 0.229). However, NAFLD was independently associated with higher Ln (CACS + 1) in the group with higher HbA1c (HbA1c ≥ 7%), after adjusting for other covariates (*P* = 0.010) (Table 3, Figure 2).

**Table 3:** **Multivariate linear regression analysis for the relationship with ln (CACS + 1) according to the HbA1c level**

**HbA1c < 7%**	**β-coefficient**	**95% CI**	***P*** **-value**
NAFLD	−0.627	−1.654-0.399	0.229
Age	0.091	0.029-0.153	0.004
Male sex	1.252	−0.126-2.629	0.075
Hypertension	0.521	−0.397-1.439	0.263
Body mass index	0.059	−0.283-0.401	0.734
Waist circumference	0.015	−0.108-0.139	0.804
HDL cholesterol	−0.014	−0.063-0.035	0.577
Triglyceride	0.006	0.000-0.013	0.059
**HbA1c ≥ 7%**			
NAFLD	1.503	0.373-2.634	0.010
Age	0.123	0.061-0.185	<0.001
Male sex	1.415	−0.203-3.034	0.086
Hypertension	0.364	−0.690-1.418	0.494
Body mass index	−0.002	−0.415-0.411	0.991
Waist circumference	0.018	−0.124-0.161	0.800
HDL cholesterol	−0.023	−0.084-0.038	0.458
Triglyceride	−0.002	−0.012-0.008	0.683

*Abbreviations*: *NAFLD* Nonalcoholic fatty liver disease, *CI* confidence interval.

## Discussion

Studies investigating the association between nonalcoholic fatty liver disease (NAFLD) and coronary artery calcium score (CACS) in diabetic patients have shown conflicting results. This study showed that NAFLD and HbA1c level had an interaction with CACS in diabetic patients. NAFLD was found to be independently associated with CACS in diabetic patients with higher HbA1c, but not in those with lower HbA1c (<7%). This is the first study demonstrating the effect modification of glycemic level on the association between NAFLD and CACS, which suggests that more strict control of hyperglycemia and more aggressive management of NAFLD could be implemented to prevent coronary atherosclerosis.

The exact underlying mechanism for the association of NAFLD and CACS in hyperglycemic conditions is not yet clear. Multiple highly interrelated factors may contribute to the relationship of NAFLD, CACS and diabetes. Some of the possible pathways are as follows [1]. First, NAFLD itself stimulates insulin resistance, leading to accelerated atherosclerosis [21]. A previous study demonstrated a linear relationship between hepatic fat content and hepatic insulin sensitivity [22]. Also, it was revealed that poor glycemic control in diabetic patients was associated with insulin resistance, as estimated by euglycemic hyperinsulinemic clamp [23]. This suggests that insulin resistance could be amplified in NAFLD patients with hyperglycemia, which may be associated with the presence of coronary atherosclerosis. Second, oxidative stress and inflammation is another link of NAFLD, hyperglycemia, insulin resistance and cardiovascular disease. Reactive oxygen species produced in hepatic steatosis induce hepatocyte injury, release cytokines, and makes pro-inflammatory milieu which progresses the liver damage of NAFLD also adding further atherogenic stimuli [7,24,25]. Higher HbA1c level has also been found to be associated with oxidative and pro-inflammatory status, having specific association with elevated TNF-α and C-reactive protein, further amplifying the atherogenic environment [23]. Third, decreased adiponectin concentrations, an adipose-secreted cytokine with anti-atherogenic properties, is also associated with NAFLD, and independently predicted cardiovascular disease in large prospective studies [1,26-28]. Hyperglycemia is also known to be associated with hypoadiponectinemia [23], therefore additional decrease of adiponectin caused by NAFLD and hyperglycemia may aggravate subclinical atherosclerosis. In addition to these mechanisms, abnormal lipoprotein metabolism might also act on the pathways of NAFLD and coronary artery atherosclerosis in diabetic patients. More studies are warranted to investigate the exact mechanism of the interaction between NAFLD and hyperglycemia in the presence of atherosclerosis.

There were some limitations in this study. First, the study population is not very large. Second, since this was a cross-sectional study, it is hard to know the causal relationship between NAFLD and CACS. A larger-scale prospective study is warranted. Third, ultrasonography was used to diagnose NAFLD without histologic confirmation of the liver, which is regarded as the gold standard for NAFLD diagnosis. However, a histologic diagnosis of NAFLD in the entire study population is difficult to accomplish, and has the risk of complications. Fourth, cautious interpretation is necessary to applying the results to the general population considering that this study includes subjects with diabetes who perform coronary CT to evaluate their cardiovascular risk. More male and subjects with elevated risk of atherosclerosis than general population might be included in this study.

## Conclusions

In conclusion, NAFLD was independently associated with subclinical coronary artery atherosclerosis in diabetic patients with higher HbA1c (≥7%) after adjustment for other risk factors, but not in diabetes patients with lower HbA1c (<7%). More attention should be given to coronary artery disease in patients with NAFLD, especially those with hyperglycemia.

## Abbreviations

NAFLD: Nonalcoholic fatty liver disease
CACS: Coronary artery calcium score
HbA1c: Glycated hemoglobin
BMI: Body mass index
WC: Waist circumference
AST: Aspartate aminotransferase
ALT: Alanine aminotransferase
GGT: Gamma-glutamyl transpeptidase
